# Comparing methods for drug–gene interaction prediction on the biomedical literature knowledge graph: performance versus explainability

**DOI:** 10.1186/s12859-023-05373-2

**Published:** 2023-06-30

**Authors:** Fotis Aisopos, Georgios Paliouras

**Affiliations:** Institute of Informatics and Telecommunications, National Centre for Scientific Research Demokritos, Athens, Greece

**Keywords:** Drug–target interaction, Knowledge graph, Graph embeddings, Deep learning

## Abstract

This paper applies different link prediction methods on a knowledge graph generated from biomedical literature, with the aim to compare their ability to identify unknown drug-gene interactions and explain their predictions. Identifying novel drug–target interactions is a crucial step in drug discovery and repurposing. One approach to this problem is to predict missing links between drug and gene nodes, in a graph that contains relevant biomedical knowledge. Such a knowledge graph can be extracted from biomedical literature, using text mining tools. In this work, we compare state-of-the-art graph embedding approaches and contextual path analysis on the interaction prediction task. The comparison reveals a trade-off between predictive accuracy and explainability of predictions. Focusing on explainability, we train a decision tree on model predictions and show how it can aid the understanding of the prediction process. We further test the methods on a drug repurposing task and validate the predicted interactions against external databases, with very encouraging results.

## Introduction

Drug development is an extremely time-consuming and expensive process with low rates of new therapeutic discoveries [1]. However, knowing targets of potential clinical significance, can play a crucial role in the process of rational drug development, as candidate compounds targeting specific proteins may be employed to achieve intended therapeutic effects (target-based drug discovery) [2]. Hence, drug–target interaction (DTI) identification has become one of the hottest research topics in the medical and pharmaceutical industry. ‘Targets’ either refer to specific proteins or related genes that have been associated with a disease. By identifying DTIs, researchers can choose pharmaceutical substances to be tested on “target” genes of interest, in the context of a clinical trial.

However, a drug rarely interacts with a single known target [3], a phenomenon that may lead to unwanted adverse effects, but also to opportunities for drug re-purposing. Therefore, the prediction of novel, unknown drug–target interactions emerges as a useful task in drug (re)discovery, as well as other related fields.

**Fig. 1:**
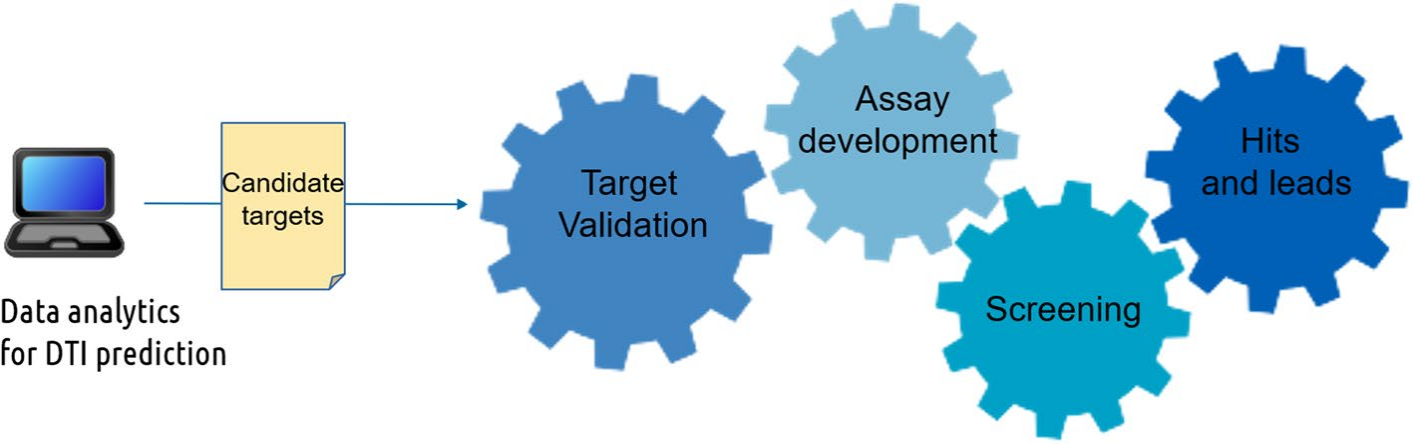
Overview of the target-based drug discovery process facilitated by computational methods. Candidate molecular targets are identified by data analytics and validated before lead discovery starts; assays and screens are then used to find a lead

Identified interactions for commercial drugs have been documented in various online databases and repositories, such as KEGG, DrugBank, ChEMBL, STITCH and others. Despite major overlaps, the DTIs existing in those repositories are neither identical, nor complete, as new, previously unknown DTIs are continuously being added. These additions may be the result of genetic [4] or proteomic research [5], or they can be generated by computational methods. While in-vivo experiments provide the ultimate validation of such interactions, genetic and proteomic validation methods are also considered adequate. However, only a limited number of DTIs can be tested in a ‘wet lab’ experiment, making such experiments impractical in terms of time and resources needed. Therefore, data analytics methods for DTI prediction can make the discovery process more efficient, by minimising the list of target candidates to be tested in a ‘wet lab’ experiment (Fig. 1).

Several recent publications (e.g. [2, 6, 7]) focus on the exploitation of Knowledge Graphs for the task of DTI prediction. In particular, this paper proposes the use of a disease-specific Knowledge Graph, generated automatically from the scientific literature, in order to identify highly probable, but yet undocumented DTIs. For this purpose, a benchmark if first created, aggregating disease-specific DTIs that are documented in existing databases. Then, a variety of machine learning methods are being tested on the benchmark, striving for a balance between predictive accuracy and explainability of predictions. The latter is particularly important, when justifying the need for a resource-intensive ‘wet lab’ experiment.

Thus, the main contributions of this work are summarized in the following points:

- A methodology and software to cross-reference drug and target identifiers across different databases and open vocabularies. Using this methodology, a benchmark is created for Lung Cancer, providing drug-gene interactions, drawn from different online repositories (DrugBank, KEGG, TTD, DGIdb). The benchmark is made publicly available.^1^
- A comparative analysis of state-of-the-art link prediction approaches for predicting DTIs on a disease-specific Knowledge Graph derived from the biomedical literature. In addition to existing methods, we introduce an explainable path analysis technique, and compare against various other methods.
- The top-50 predictions of unknown DTIs produced by the best methods are cross-validated against online repositories, with very encouraging results.

The rest of the document is organised as follows:  “Related work” section presents recent work on link prediction in biomedical Knowledge Graphs, and explainability of such approaches. “Generating a knowledge graph from biomedical literature” section briefly describes the Biomedical Literature Knowledge Graph construction pipeline, while  “Methods” section analyses the various approaches being assessed, including a new path-based discovery method (BLGPA). Lastly, “Evaluation” section evaluates the performance of different approaches and discusses explainability of the predictions, while  “Drug rediscovery test” section presents a drug re-positioning test for Lung Cancer, identifying and cross-checking unknown disease-specific DTIs. Finally, “Conclusions” section summarizes the presented work, indicating open research issues.

## Related work

There is rich literature related to computational methods for the prediction of drug–target interactions. Recent work mainly uses deep learning techniques on drug or protein descriptors (e.g. [8–10]), yielding encouraging results. Focusing on approaches that treat DTI prediction as a link prediction problem in Knowledge Graphs, we distinguish the following categories of methods.

### DTI prediction on biomedical knowledge graphs build from open databases

SemEP [11] and esDSG [12] adopt similar approaches, extracting certain relations (i.e. drug–target interactions, drug-drug and target-target similarities) from open ontologies to produce a low-dimensional graph, and then apply edge-based community detection. Those approaches employ edge partitioning for community detection to identify missing drug–target protein interaction links. The proposed methods are validated either on benchmark datasets (Ding et al. [13]) or by using external DTI databases (STITCH, KEGG) as gold standards. On the other hand, Mohamed et al. [2] present an approach that constructs a KG from various chemical and protein-related databases (Drugbank, KEGG, InterPro, Uniprot). Using this KG, they propose a graph embedding model (TriModel) to learn vector representations for drugs and targets. Trimodel is a tensor factorization method, based on DistMult and ComplEx, and can be used to predict interaction links between drugs and proteins. The prediction process uses confidence scores that are calculated directly by the embedding models’ scoring function. Similarly, Ye et al. [7] propose a framework called KGE_NFM that combines Knowledge Graph embeddings and recommendation system techniques for DTI predictions. The authors emphasize the cold start problem of new protein targets being identified for a complicated disease. KGE_NFM captures the latent information from KGs using graph embeddings (KGE) and then applies a neural factorization machine (NFM) to extract features for the DTI prediction task.

### Graph convolutional networks

Relational Graph Convolutional Networks (RGCNs) [14] extend Graph Convolutional Networks to deal with the data of heterogeneous Knowledge Graphs that contain various relation types [15].

Following such an approach, Zitnik et al. [16] present a method called Decagon that models polypharmacy side effects in a drug–protein Knowledge Graph. The use case examined in that paper focuses mainly on drug-drug interactions, but it can be extended to DTIs. An inductive version of RGCN (I-RGCN) is proposed in [17] and is used to discover potential treatments for COVID-19. The method is applied on a biological Knowledge Graph, termed Drug Repurposing Knowledge Graph (DRKG), including information from six existing databases (DrugBank, Hetionet, GNBR, String, IntAct and DGIdb).

### Link prediction on biomedical literature graphs

The method that is closest to the work presented in this paper is SemaTyP [1], which analyses PubMed articles using SemRep [18] and extracts semantic features from the resulting Knowledge Graph paths. The goal of SemaTyP is to predict interactions between entity triplets (drug–protein-disease). Triples are generated and scored against TTD^2^ that is used as a gold standard. The best scoring triples are then selected for Drug Discovery. The successor of SemaTyP, GrEDeL [6], constructs a similar KG, and then calculates the TransE embeddings of all entities/relations along each path, using them to train an LSTM network model, in order to evaluate candidate DTI pairs.

In our earlier work, we have also proposed a method, called DDI-BLKG[19], which uses simple path analysis (frequent relational chains), in order to identify previously unknown drug-drug interactions. In the experimental comparison of this paper we include an extended version of DDI-BLKG, called BLGPA, focusing on DTI prediction.

### Link prediction explainability

As mentioned in “Introduction” section, for any prediction to find its way to the wet lab, trust by health science researchers is essential. Therefore, statistical and traditional machine learning methods based on rules [20] and using features that make sense to humans [21] have an advantage over complex black-box models. For the more complex approaches, explainability techniques such as LIME (Local Interpretable Model-Agnostic Explanation) [22] are needed, in order to gain an understanding of which features are used and how for making a decision. However, graph embeddings model the nodes and links of KGs with derived features that are hard to interpret, thus requiring special explainability approaches.

Various methods have been proposed to explain the link predictions of an RGCN, with limited success, regarding the quality of explanations, i.e. how understandable they are in fact by the prediction consumers. The most prominent one is ExplaiNE [23], which quantifies how the predicted probability of a link changes when weakening or removing a link with a neighboring node. One the other hand, GNNExplainer [24] explains the predictions of any GNN by learning a mask over the adjacency matrix, in order to identify an informative subgraph, together with a small subset of node features, that are most influential for the prediction. Halliwell [25] recently attempted to evaluate such explainability techniques, proposing the use of a scoring metric on benchmark datasets, which allows comparison of their explanations.

## Methods

### Generating a knowledge graph from biomedical literature

MEDLINE and PubMed are the most popular sources of biomedical knowledge, offering more than 28 million citations. PubMed supports semantic retrieval based on manually curated topic annotations using the MeSH vocabulary.^3^ Additionally, PubMed Central (PMC) provides the full text of about 4.7 million articles. In this work, we aim at generating disease-specific Knowledge Graphs from biomedical literature, exploiting publicly available tools to retrieve relevant article abstracts as well as their full text when available.

**Fig. 2:**
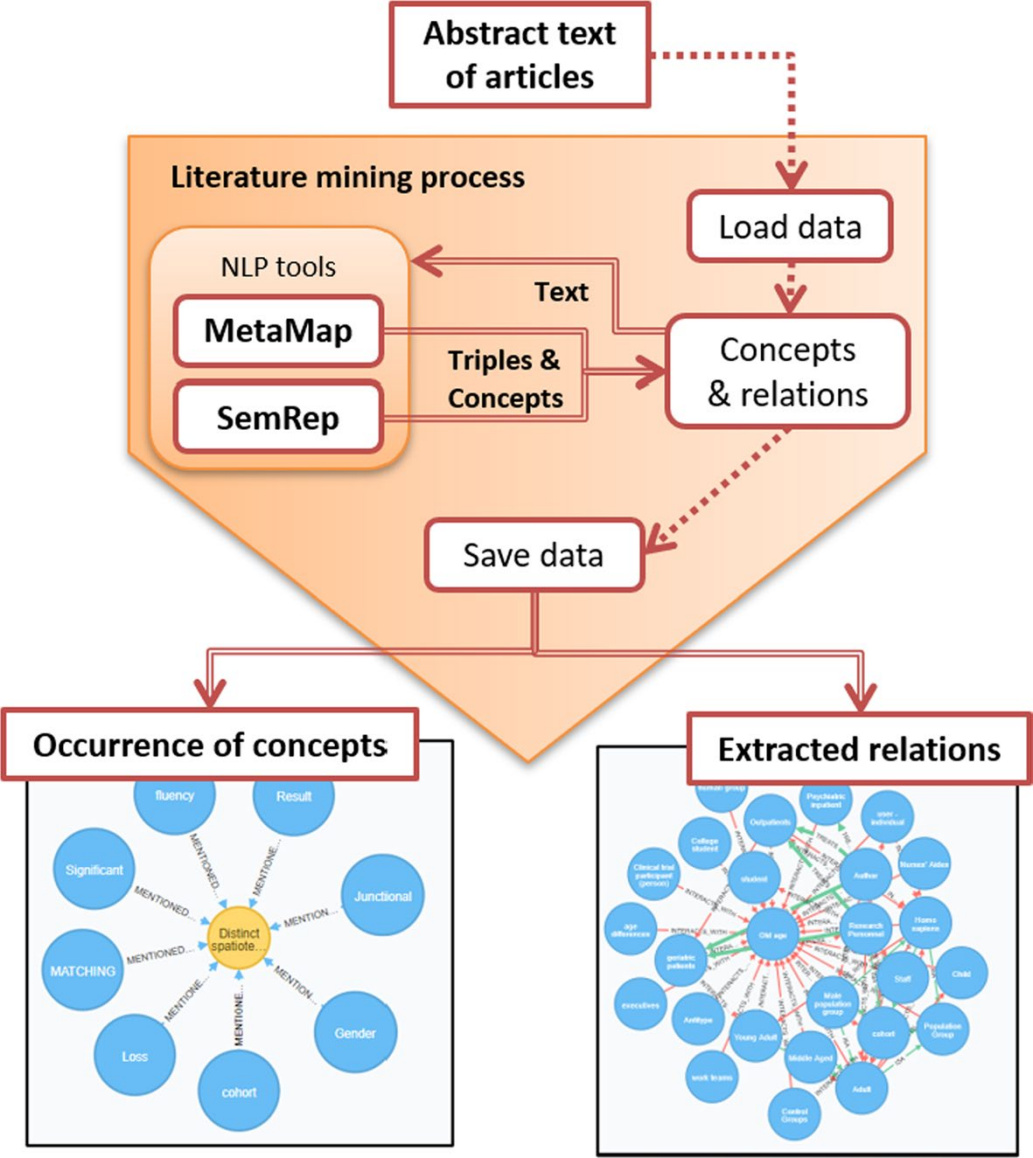
Overview of the process of harvesting and analysing biomedical literature text, using Natural Language Processing tools

As shown in Fig. 2, all articles relevant to a particular disease (e.g. Lung Cancer) are harvested through appropriate semantic queries to PubMed and the title, abstract and topic annotations for each article are extracted [26]. In addition, PMC is queried and any available full text for the articles is also retrieved.

The textual information collected in this manner is then mined to identify semantic entities and their relations. The basis for the conceptual modeling of information is the Unified Medical Language System (UMLS) vocabulary [27], and in particular the Metathesaurus [28], which links differing expressions of the same biomedical meaning under a single *concept*, and the Semantic Network [29], which enriches the concepts, grouping them into 133 semantic types, such as diseases and chemicals, and defines semantic relations between those types.

In order to harness the power of this representation, SemRep  [18] is being employed, a UMLS-based tool that extracts biomedical predications, i.e. semantic triples in the form of subject-predicate-object, from unstructured text. The subject and object arguments of these predications are concepts from the UMLS and the predicate is one of the semantic relations of the Semantic Network, connecting the semantic types of the subject and object, in the context of the specific sentence (Literature mining process of Fig. 2).

In addition to the relations defined by the Semantic Network, we create two new types, which we call “*MENTIONED_IN*” and “*HAS_MESH*”. The “*MENTIONED_IN*” relation expresses the occurrence of a concept in an article, as can be seen in Fig. 2. The motivation behind this new relation is that it allows us to take into account the co-occurrence of concepts in the documents. Co-occurrence provides a way of measuring the association between two terms and potentially helps in targeting interesting associations (for example between medications and disorders). Articles also appear in the knowledge graph as nodes connected with concepts through “*MENTIONED_IN*” edges.

On the other hand, the “*HAS_MESH*” predicate allows us to capture the semantic information generated by the curators of biomedical articles. Each article in PubMed is associated with a set of MeSH tags and these associations are encoded in the graph in the form of “article-HAS MESH-concept” triples. These triples express the topics to which each article is connected, according to human curators, hence providing rather robust knowledge.

Beyond literature, ontologies and databases are other important sources of domain knowledge in the field of biomedicine. In order to enrich the information in the disease-specific KG, we have developed a harvester to integrate taxonomic relations (*is_a*) from ontologies available in the Open Biomedical Ontologies (OBO) format [30] (e.g. Gene Ontology, Disease Ontology).

The resulting disease-specific Knowledge Graph is stored in a Neo4j^4^ graph database, capturing naturally the connectivity between entities and facilitating efficient retrieval and analysis.

### Predicting drug target interaction links

Using the multi-relational disease-specific Knowledge Graph (KG) created as explained in “Generating a knowledge graph from biomedical literature” section, we aim to infer probable interactions between drugs and potential targets. Specifically, given a pair of graph nodes, corresponding to a drug and a target gene, drug–target interaction prediction can be formulated as a link prediction problem. The prediction task is modeled as a supervised learning problem, where data samples are generated from node pairs in the KG and the goal is to determine which node pairs should be linked with an interaction relation.

In this section, we present the different KG link prediction methods that we have included in our experiments. They range from simple, but explainable path classifiers to powerful, but complex Deep Learning (DL) techniques, such as Graph Convolutional Networks. Note that all methods make a closed world assumption (CWA), where a non-existing triple in the training data is interpreted as a negative interaction, i.e., the corresponding relationship is considered false [31].

#### Rule-based methods

Rule-based approaches for Knowledge Graph link prediction have an important advantage, which is their ability to provide an explanation in terms of the rules used in a prediction.

From this family of methods, we have chosen to use Anytime Bottom Up Rule Learning (AnyBURL) [20], an efficient approach to learning logical rules from large Knowledge Graphs. AnyBURL generalizes positive examples, which take the form of paths between drugs and their targets, to generate rules that are applicable to other cases and can infer missing graph links. The approach is similar to the path ranking algorithm (PRA) [32], learning path rules and ranking them according to an estimated level of confidence. In the context of this work, AnyBURL has been configured to provide the confidence score for each candidate interacting pair of nodes.

#### Semantic path analysis

As mentioned in “Related work” section, Semantic Type Path (SemaTyP) is another explainable technique for DTI prediction. SemaTyP analyses a biomedical Knowledge Graph, called SemKG, produced from all abstracts of PubMed posted before June 2013, in order to address the following tasks:

1. Classify all paths from Drug nodes passing through Target nodes to end at Disease nodes, (DTD paths). The goal is to identify the paths with interacting/associated (drug–target-disease) triples.
2. Rediscover drugs that are suitable for a disease, based on the DTD path scores.

The DTD path scoring task entails the DTI task examined in the current work, under certain assumptions. Specifically, for the training data, SemaTyP focuses on paths of the form $\pi ^l = \rho (drug_i \Rightarrow disease_i; target_i, l)$; denoting paths of length *l*, reaching node $disease_i$ from source node $drug_i$, over node $target_i$. A set of features are extracted from each path, by aggregating the occurrences of subject/object node semantic types and the types of different relations along the path. A logistic regression classifier is then employed, learning to discriminate positive pair paths from the negative ones.

#### A hybrid technique: BLGPA

In our earlier work, we presented a semantic path encoding method akin to SemaTyP, called DDI-BLKG. Here, we extend that method with a simple Path Ranking approach, leading to a hybrid link prediction technique, which we call Biomedical Literature Graph Path Analysis (BLGPA).

**Fig. 3:**
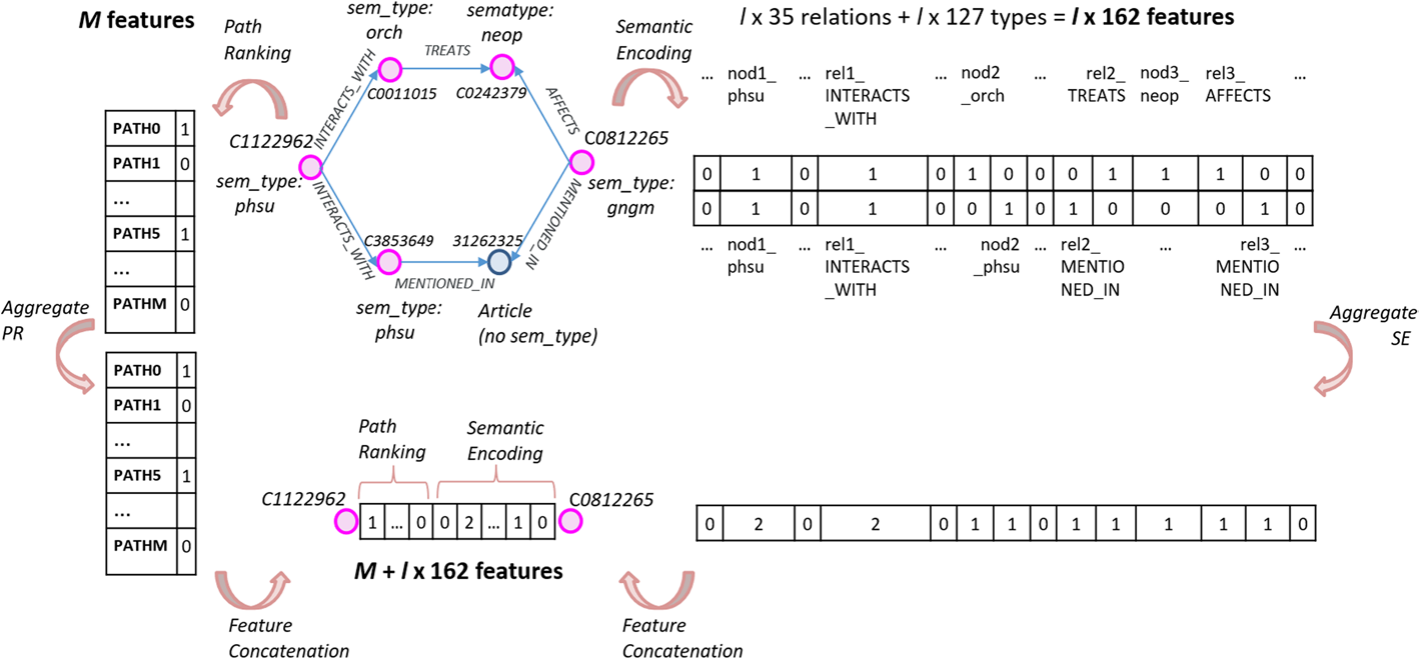
Overview of the BLGPA feature extraction process, forming the final feature representations of drug–target pairs. Note that the chain of relations *(INTERACTS_WITH,TREATS,AFFECTS)* is represented in the path list as PATH0 and *(INTERACTS_WITH,MENTIONED_IN,MENTIONED_IN)* as PATH5

BLGPA is trained on data samples generated by aggregating all possible paths between a specific drug-gene pair of nodes. More formally, let $V = {v_1, v_2,..., v_N}$ be the set of nodes of the graph, consisting of the biomedical entities, mapped to UMLS concepts, along with the articles they were mentioned in. Additionally, let $R = {r_1, r_2,..., r_M}$ denote the set of relations in the graph and (*d*, *t*) a drug-gene pair under examination. Also, let $\pi ^l$ be a single path of length *l* connecting *d* and *t*. The path $\pi ^l$ consists of a sequence of nodes and relations starting from node *d* and ending at node *t*: $(d,r_0,v_1,r_1,v_2,r_2,...,r_{l-1},t)$. Let $\Pi _{d,t} = \{\pi _1^l, \pi _2^l,..., \pi _{N_{(d,t)}}^l\}$ be the set of all possible paths of length up to *l* between the examined pair of nodes, as illustrated in Fig. 3. Each path $\pi _i^l \in \Pi _{d,t}$ is processed in order to extract two types of features:

- *Semantic Encoding (SE)*
- *Path Ranking (PR)*

*Semantic Encoding - SE.* Given a path $\pi _i^l = (d,r_0,v_1,r_1,v_2,r_2,...,r_{l-1},t)$ of length *l* between a drug *d* and a gene *t*, a straightforward feature representation is to encode the involved node and relation types. MetaMap uses 127 different semantic types^5^$ST=\{st_1,st_2,...,st_{127}\}$, in its entity recognition process, while from the UMLS Semantic Network^6^ we have used the 35 most relevant and expressive semantic relations $SR=\{sr_1,sr_2,...,sr_{35}\}$. In order to preserve the order in which node and relation types appear in a graph path, we generate $127+35=162$ features for every possible position *j* in the path sequence $\pi _i^l$: $fv_{SE,j}=\{nod\textit{j}_{st_1},nod\textit{j}_{st_2},...,nod\textit{j}_{st_{127}},rel\textit{j}_{sr_1},rel\textit{j}_{sr_2},...,rel\textit{j}_{sr_{35}}\}$, where $nod\textit{j}_{st_x}$ denotes one of the 127 possible node types and $rel\textit{j}_{sr_y}$ one of the 35 relation types. Each feature captures the frequency of a semantic type or a relation at a specific position in the path, i.e. the number of different articles from which the corresponding triplet with those types has been extracted. Note that the subject/object nodes of the same triplet may be attributed to different semantic types in different articles. Given a maximum path length *l*, the full SE feature vector of a given path will have a length of: $|fv_{SE}|=l\times 162$, ignoring the semantic type of the final node, which is always ‘gngm’: Gene or Genome. Given the above, for a specific path $\pi _i^l$ the corresponding SE feature vector will be: $fi_{SE}^{(d,t)} = \underset{n=0}{{\mathop {\Vert }\limits ^{n=l}}}[c_{st_1}, c_{st_2},..., c_{st_{127}}, c_{sr_1}, c_{sr_2},..., c_{sr_{35}}]^T$ where $c_{st_i}$, $c_{sr_i}$ denote the frequency count of the specific semantic type and semantic relation respectively, and $\Vert$ denotes the concatenation of these occurrence vectors for each transition in the path. For paths $\pi _{(d,t)}^n$ of length $n<l$ the last $l-n$ components of the vector are set to zero.

Since we are interested in representing each drug-gene pair with a single feature vector, we aggregate the feature vectors of the different paths leading from *d* to *t* by element-wise summation of the corresponding feature vectors. Thus, the SE feature vector for each pair will be:

1 $$\begin{aligned} fv_{SE}^{(d,t)} = \sum _{i=1}^{N_{(d,t)}}fi_{SE}^{(d,t)} \end{aligned}$$

*Path Ranking - PR.* In contrast to semantic encoding, path ranking features focus on the relations along a graph path, ignoring node types. Hence, a path of length *l* is simply a sequence of relation types: $\pi _i^l = (r_0,r_1,r_2,r_3)$. The idea behind this formulation is that we are not focusing our interest in the specific biomedical concepts that are found in the paths, but rather in the chain of relations that one must traverse to get from the starting drug node to the end gene node.

Using the labeled training data, we calculate the *support* of each relational chain, i.e. the frequency of the path, in positive and negative pairs. Based on their support, we select the M most important paths ${f_{PATH_1}, f_{PATH_2},..., f_{PATH_M}}$, with the importance score being calculated from their frequency in positives minus their frequency in negatives. Having selected these relational chains that act as possible indications for interacting pairs, we count the number of occurrences of each such chain in the paths leading from a drug node to a gene node. More formally, let all possible paths of maximum length *l* between a drug *d* and a target *t* be $\Pi = \{ \pi _1^l, \pi _2^l,..., \pi _{N_{(d,t)}}^l \}$. They are represented with a single feature vector

2 $$\begin{aligned} fv_{PR}^{(d,t)} = [c_{f_{PATH_1}}, c_{f_{PATH_2}}, ..., c_{f_{PATH_M}}]^T \end{aligned}$$

comprising the occurrence count of each $f_{PATH_i}$ in the set of paths $\Pi$, i.e.,

3 $$\begin{aligned} c_{f_{PATH_i}} = \sum _{j=1}^{N_{(d,t)}}\mathbbm {1}(PATH_i, \pi _j) \end{aligned}$$

where $\mathbbm {1}(PATH_i,\pi _j) = 1$ if the chain of relations of $PATH_i$ occurs in path $\pi _j$ and 0 otherwise. Therefore, each drug–target pair is represented by a feature vector $fv_{PR}^{(d,t)}$ of length *M*, where each feature measures the frequency of the corresponding relational chain $f_{PATH}$, as can be seen in the example of Fig. 3.

*Feature aggregation.* In order to combine the expressiveness of the two feature sets presented above, we concatenate the corresponding vectors.

Figure 3 provides an example of the feature extraction process for drug Gefitinib (CUI: C1122962) and the ERBB3 Gene (CUI: C0812265), connected by paths that are analysed to extract SE and PR features. On the left hand side, the paths illustrated include the chain of relations (INTERACTS WITH,TREATS,AFFECTS) that is represented in the important path list as PATH0 and (INTERACTS WITH,MENTIONED IN,MENTIONED IN) that is represented in the important path list as PATH5. Therefore, the aggregated PR feature vector has a value of 1 for those two paths and 0 for the rest. On the right hand side, the SE feature vector for each path has a value of 1 for each a node/relation semantic type existing in the path at this position. The aggregated SE feature creates a sum of those element values for each pair. For example, the value 2 of the feature *rel1_INTERACTS_WITH* denotes that two different paths included the *INTERACTS_WITH* relation in position 1. The bottom part of the figure illustrates the feature aggregation process, by concatenating those two aggregate feature vectors into a final vector of $M + l * 162$ features.

The resulting feature vector can be used downstream to train a classifier, in order to discriminate interacting and non-interacting drug-gene pairs. In the current scenario, we have chosen a Random Forest, as this seems to yield better results.

#### Graph embeddings

As explained in “Related work” section, Graph Embeddings (GEs) constitute a popular approach of transforming knowledge graphs to numeric vectors. Such a transformation facilitates the process of link prediction, as the numeric embeddings can be used to probabilistically infer missing edges from the existing structure of a KG. TransE is one of the most popular GE methods, modeling graph relationships as translations operating on the low-dimensional embeddings of the entities. TransE performs linear transformations, aiming at minimizing the energy function $E=|e_h+e_r-e_t|$, where $e_h,e_r,e_t$ represent the embeddings of the head, relation and tail of every triple (*h*, *r*, *t*).

In the context of this work, we have experimented with the TransE [33], DistMult[34], HoLE[35] and RESCAL[36] graph embedding models. For the downstream task of link prediction, we can follow two different approaches:

(a) The probability of a candidate interaction relation $(d,r_i,t)$ can be estimated directly from the embeddings of the corresponding pair of drug-gene nodes ($e_d, e_t$) and the embedding of the relation type ($e_{r_i}$).

(b) The node and relation embeddings of a set of training triples can be used as training features in a machine learning classifier, in order to learn discriminative patterns between positive and negative cases. In this case, the feature vector for the classifier consists of the concatenation of the embedding vectors for each candidate triple $(d,r_i,t)$:

4 $$\begin{aligned} fv_{GE}^{(d,t)}=e_d \mathbin \Vert e_{r_i} \mathbin \Vert e_t \end{aligned}$$

where $fv_{GE}^{(d,t)}$ is the concatenated feature vector of each candidate triple $(d,r_i,t)$, with $e_{r_i}$ being the embedding vector of the ‘INTERACTS_WITH’ relation, which is the same for every pair.

#### Relational graph convolutional networks

As noted in “Related work” section, Relational Graph Convolutional Networks (RGCNs) can handle the multi-relational nature of knowledge graphs, and can be utilised for downstream tasks, such as link prediction. The main components of an RGCN are [16]:

- an encoder: a graph convolutional network, operating on the Knowledge Graph and producing embeddings for all nodes;
- a decoder: a tensor factorization model, using the aforementioned embeddings to model drug-gene node interaction relations.

The encoder maps each entity $v_i \in V$ to a real-valued vector $e_i$ of dimension *m* through a function $enc: V \rightarrow \mathrm{I\!R}^m$. It initially considers the id of each node as a feature, as no other node features are included in the Knowledge Graph. Then, it aggregates information from the feature vectors of the node’s neighbors, and their own neighbors, effectively convolving information across the n-th order neighborhood, where n is the number of successive operations of convolutional layers in the neural network. For this purpose, we follow the propagation model defined by Schlichtkrull et al. [15] for calculating the forward-pass update of each node:

5 $$\begin{aligned} h_i^{(n+1)} = \sigma \left( \sum \limits _{r \in R} \sum \limits _{j \in N^r_i} \frac{1}{c^{ij}_{r}} W^{(n)}_r h^{(n)}_j + W^{(n)}_0 h^{(n)}_i\right) \end{aligned}$$

where $h_i^{(n)} \in \mathrm{I\!R}^{d^{(n)}}$ is the hidden state of node $v_i$ in the *n*-th hidden layer of the neural network, with $d^{(n)}$ being the dimensionality of this layerâ€™s representations and $N^r_i$ denoting the set of neighbor indices under relation $r \in R$. In Eq. (5), $c^{ij}_{r}$ and $W^{(n)}_0$ denote two symmetric problem-specific normalization constants that are equal to $\sqrt{|N^r_i||N^r_j|}$ and $1/|N^r_i|$ respectively, and $W^{(n)}_r$ a trainable relation-type specific weight matrix.

On the other hand, the decoder of the RGCN reconstructs the edges of the graph relying on the vertex representations, by scoring triples through a function $dec: \mathrm{I\!R}^m \times R \times \mathrm{I\!R}^m \rightarrow \mathrm{I\!R}$, with *m* being the dimension of the encoder embedding model. As in the original RGCN model, we use the DistMult factorization as the link scoring function in the decoder. In DistMult, every relation *r* is associated with a diagonal matrix $R_r \in \mathrm{I\!R}^{m \times m}$ and a each candidate triple $(d,r_i,t)$ is scored as follows:

6 $$\begin{aligned} S(d,r_i,t) = e^T_{d} R_{r_i} e_{t} \end{aligned}$$

where $e_{d}, e_{t}$ are the embeddings of nodes *d* and *t* respectively, and $R_{r_i}$ the diagonal matrix of the ‘INTERACTS_WITH’ relation. Thus, the score $S(d,r_i,t)$ provided by the decoder outputs a prediction probability for a candidate triple $(d,r_i,t)$ through a factorized operation.

## Results

### Evaluation

#### Benchmark dataset and evaluation metrics

In order to compare objectively the different methods that we presented in Results section, we compiled a DTI benchmark dataset, drawing ground-truth data from various online databases. In this process, we have avoided information that is only generated automatically, through text mining, and focused on most trustworthy sources, namely DrugBank [37], KEGG [38], DGIdb [39] and TTD [40] data. Additionally, we implemented two side-projects to identify and cross-check drug and gene relations across various databases and open vocabularies:

- A methodology^7^ for cross-matching drug identifiers in different databases and vocabularies. This has resulted in a mapping^8^ of drug ids from Drugbank, STITCH, UMLS, KEGG, PubChem, ChEMBL, ChEBI, CAS Registry, bindingDB, ZINC, and TTD.
- A methodology^9^ for cross-matching protein target identifiers in different databases and vocabularies. This has resulted in a different mapping^10^ of gene/protein ids from Uniprot, DisGeNET, Ensembl, and TTD.

The union of DrugBank, KEGG, DGIdb and TTD provided 44,169 positive drug-gene interactions in total, with 1931 of those related on one side (drug) or the other (gene) to Lung Cancer (LC), the disease used as a case study in our work. Regarding the negative drug-gene pairs, we extracted 627,971 pairs, for which no interaction is reported in any of the above databases. Additionally, since the Knowledge Graph described in “Generating a knowledge graph from biomedical literature” section contains biomedical entities in the form of UMLS identifiers, the drugs and genes included in the benchmark had to be represented by their respective Concept Unique Identifiers (CUIs). However, only a subset of those CUIs is present in our disease-specific KG. The remaining pairs could not be used in our experiments. Therefore, the final LC benchmark dataset comprised the data shown in the last row of Table 1. It is worth noting that, using our methodology, one can easily generate similar benchmarks for other diseases of interest.

**Table 1:** Statistics of the benchmark dataset derived from DrugBank, KEGG, DGIdb and TTD

	Positive	Negative	Total
Drug gene pairs	44,169	583,802	627,971
LC-related drug gene pairs	1931	583,802	585,733
CUI pairs in KG	1538	82,778	84,316

Utilising this dataset the different link prediction methods were assessed, using 10-fold Cross Validation (CV). The macro-average values of Precision, Recall and F1-Score for the positive class (drug-gene interactions) were used as the main evaluation metrics in the comparative evaluation.

#### Data imbalance

**Fig. 4:**
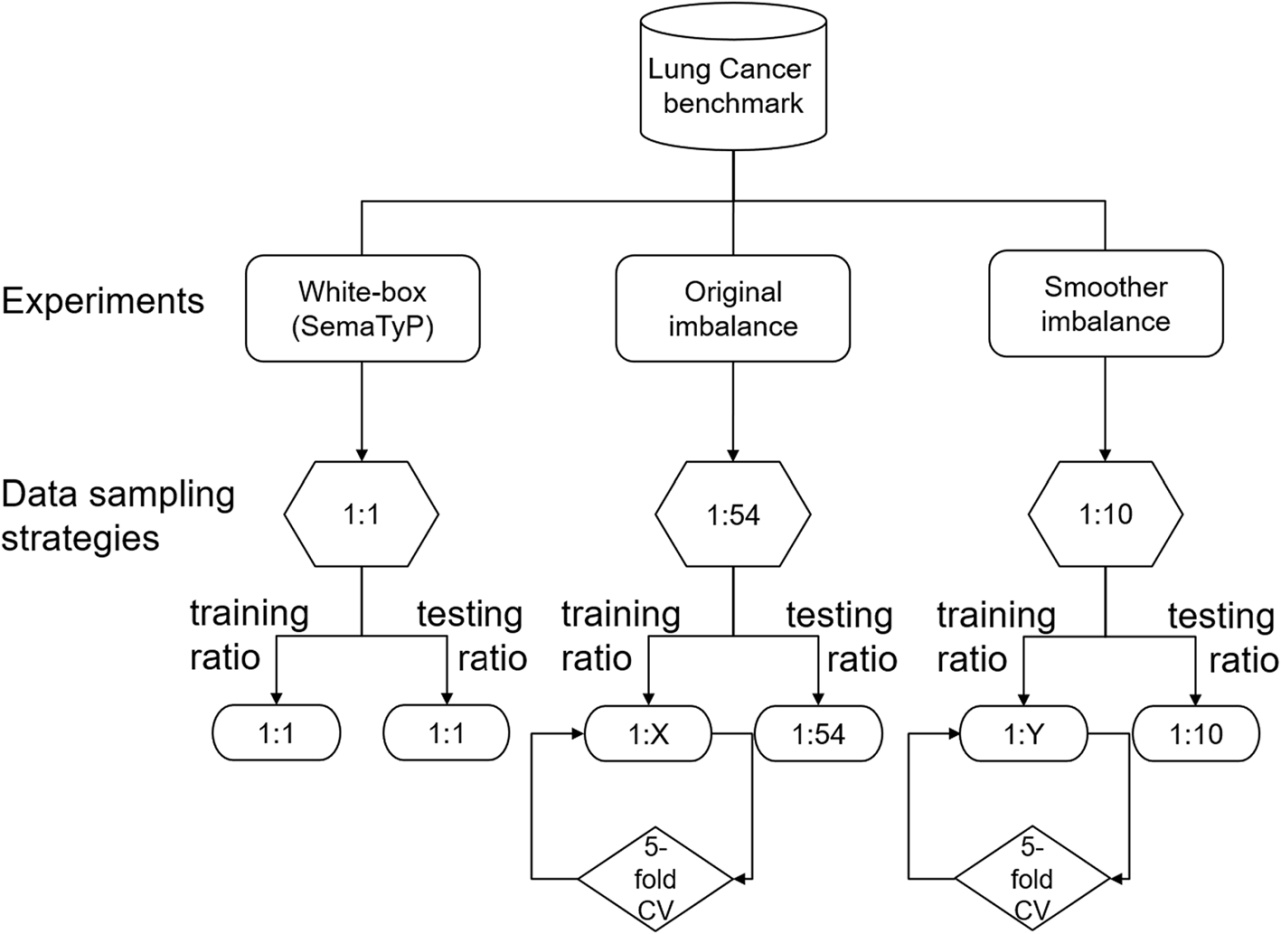
Overview of the different sampling steps and strategies for the experiments performed

As shown in Table 1, the positive-to-negative ratio is roughly 1:54. Since SemaTyP was originally tested on a balanced set [1], we also opted for a balanced sampling strategy (1:1) in our initial experiments. However, we have also tested all black-box methods at the 1:54 ratio, and at a 1:10 ratio, in order to experiment with a smoother imbalance. Figure 4 presents the different sampling strategies, resulting in different experimental set-ups. In the two imbalanced cases, we have further subsampled the negative class in the training set of each fold, by running an inner 5-fold cross validation and selecting the best ratio (denoted in Fig. 4 as 1:X and 1:Y respectively).

#### Configuration of the methods

In this subsection, we present the main design choices of our experiments, as well as the configuration of important parameters of the methods being compared.

**AnyBURL**: In order to directly compare white-box methods, we use a balanced dataset of drug-gene interactions. However, for each positive training triple, AnyBURL requires a set of negative triples (denoted by the hyper-parameter *UNSEEN_NEGATIVE_EXAMPLES*) during its learning process, in order to be trained to identify DTI pairs. Thus, in the context of these experiments, one negative triple is provided for each positive triple. Also, we have increased the learning period to 5000 s, in order to obtain a large set of rules. For the remaining hyper-parameters (*TOP_K_OUTPUT*, *THRESHOLD_CORRECT_PREDICTIONS*), we have used the default values suggested for large scale settings.

**SemaTyP**: Due to the size of our Knowledge Graph, we opted for relatively short paths. Specifically, we noticed that connecting drug–target-disease triplets with paths longer than 4 led to an explosion of possibilities. Therefore, we opted for DTD paths of maximum $l=4$, thus including drug-gene paths of maximum length 3. As suggested in the original SemaTyP experiments [1], a balanced sample of 1,538 DTD triplets for each class was used (corresponding to 1,538 drug-gene pairs per class). To evaluate SemaTyP we used 10-fold CV over a Logistic Regression classifier. The Logistic Regression model achieves higher performance with L2 regularization, setting the parameter $\lambda 2=1.0$. Different from the original SemaTyP paper [1], which assesses path classification performance, we calculated the performance of classifying (drug–target-disease) triplets, which correspond to DTI predictions, by aggregating semantic features at the triplet level. This was done by extending the SemaTyP algorithm, in order to sum feature values for all different paths of a single triplet.

**BLGPA**: For BLGPA, we determined experimentally that the maximum allowed path length does not need to be higher than 3, i.e. $l=3$. Longer paths led to worse results, due to the interconnectedness of the graph. This happens because there are strong hub nodes in the graph, defining basic concepts of the domain. Such hubs are reachable from almost every other node in the KG in a few hops. This, in turn, implies that all nodes are connected to each other within a few hops and longer relational chains do not lead to meaningful patterns. Moreover, the number of top-ranked paths was set to $M=100$ (the list of selected paths is provided in the Additional file 3), as more features did not add significant value to the classifier’s training. Thus, the resulting feature vectors of BLGPA have a total length of $l\times 162 + M = 3 \times 162 + 100= 586$ elements. Link prediction was achieved by a Random Forest classifier, using default settings and 100 estimators.

**Graph embeddings**: Following experimentation, we have opted for embeddings of size=100, training for 100 epochs with an early stopping option. As discussed in Graph embeddings” section, two approaches have been investigated for link prediction using GEs:

(a) Using directly the graph embeddings. The graph embedding models were evaluated in a 10-fold CV scheme. At each repetition, 90% positive drug-gene relations were included in the Knowledge Graph with a new label called ‘Interaction’. The new graph was used to estimate node and relation type embeddings. The remaining 10% of the ground-truth positive and negative drug-gene relations were used as test data, based directly on the triple score of the embedding model. The results that we obtained using this approach were very low and are not presented here.

(b) Using an additional classifier. We generated the node and relation type embeddings and used these to train a separate classifier in a supervised manner. As ground-truth, we used again the 90% of ‘Interacts’ relations from our benchmark dataset pairs per fold.

**Relational graph convolution network**: For the RGCN, we have used an Encoder with 100 hidden layers and a DistMult decoder. In the original (1:54 ratio) sampling scenario the RGCN model was trained for 50 epochs, while in the (1:10 ratio) scenario only for 15 epochs, in order to avoid overfitting in both cases.

#### Performance results

***White-box methods.***

Three of the methods presented in “Methods” section (SemaTyP, AnyBURL, and BLGPA) generate models that are traceable, meaning that their DTI predictions can be explained in terms of the characteristics of the drug and its potential target. For this reason, we call these “white-box” methods and their predictive accuracy on the graph that we have constructed is shown in Table 2. As can be observed, the rule-based technique AnyBURL has achieved significantly lower accuracy results. The BLGPA method was evaluated with and without Path Ranking (PR) features, as well as with Feature Selection (FS), based on importance weights,^11^ not improving, however, its performance. As highlighted in Table 2, BLGPA with PR features achieves the best F1-Score.

**Table 2:** Comparison of white-box methods on the task of DTI prediction, using 10-fold cross-validation on a balanced positive-to-negative ratio (1538:1538=1:1). Maximum length for SemaTyP DTD paths was set to $l=4$

Metric	AnyBURL	SemaTyP	BLGPA	BLGPA PR	BLGPA PR+FS
Precision	0.86	0.84	0.90	0.90	0.90
Recall	0.44	0.80	0.88	0.89	0.89
F1-Score	0.58	0.82	0.89	**0.90**	0.89

The first column of Table 2 provides the results of SemaTyP on the task of path classification, while the second column on triple classification. The performance improvement of BLGPA over SemaTyP for triple classification is a result of the enriched feature set, which contains also Path Ranking (PR) features, as well as the additional relation types (co-mentions, and MeSH topic relations).

***Black-box methods.*** Moving to the graph embedding approaches, whose predictions are harder to explain, Tables 3 and 4 present DTI class prediction accuracy of the various methods, highlighting the best F1-score achieved. BLGPA is also included for comparison. Results on the original ratio 1:54 are presented in Table 3 and for 1:10 in Table 4. As in white-box methods comparison, the BLGPA method has been evaluated with and without Path Ranking (PR) features, as well as with Feature Selection (FS), based on importance weights.

**Table 3:** Comparison of graph embedding methods and BLGP, using 10-fold cross-validation on the original positive-to-negative ratio (1538:82778=1:54)

Metric	BLGPA	BLGPA PR	BLGPA PR+FS	RGCN	TransE	DistMult	HolE	RESCAL
Precision	0.60	0.65	0.62	0.67	0.73	0.65	0.72	0.71
Recall	0.56	0.54	0.58	0.75	0.63	0.63	0.63	0.50
F1-score	0.58	0.59	0.60	**0.71**	0.68	0.64	0.68	0.59

**Table 4:** Comparison of graph embedding methods and BLGP, using 10-fold cross-validation on the reduced positive-to-negative ratio (1538:15380=1:10)

Metric	BLGPA	BLGPA PR	BLGPA PR+FS	RGCN	TransE	DistMult	HolE	RESCAL
Precision	0.77	0.79	0.81	0.83	0.86	0.73	0.81	0.84
Recall	0.70	0.70	0.69	0.85	0.85	0.85	0.85	0.70
F1-score	0.73	0.74	0.75	0.84	**0.85**	0.79	0.83	0.76

In terms of F1-score, the RGCN and TransE models seem to outperform the others. Furthermore, the RGCN method seems to reach higher recall, while the TransE model higher precision in both cases. Let us note that a lower precision is expected to some extent, due to the fact that some False Positives may be in fact correct predictions.

#### Efficiency comparison

Training any of the aforementioned graph embedding models on a GPU server, having an NVIDIA RTX A6000 Graphics Card with CUDA Version: 11.4, requires 409.48 s/epoch on average. Due to the early stopping option selected, the training process usually stops at around 50 epochs. Hence, the training and testing of each fold takes approximately 5.69 h. On the other hand, RGCNs built with PyTorch geometric require 33 s/epoch on average on the same server and settings. This corresponds to a total of 0.46 hrs to train the model for 50 epochs and test it in each fold.

On the other hand, the cost of the BLGPA method, being the most competitive of the white-box approaches, depends mainly on the cost of path retrieval. This cost has been calculated 0.83 s/pair on average on the same machine. This corresponds to 19.45 h for all CUI pairs, with the cost of training and testing the random forest classifier being minimal. It is also worth-noting that BLGPA does not benefit from a GPU server and can be ran at a similar cost on an Ubuntu server with 12GB RAM and 8 CPU cores.

#### Explaining BLGPA predictions

**Fig. 5:**
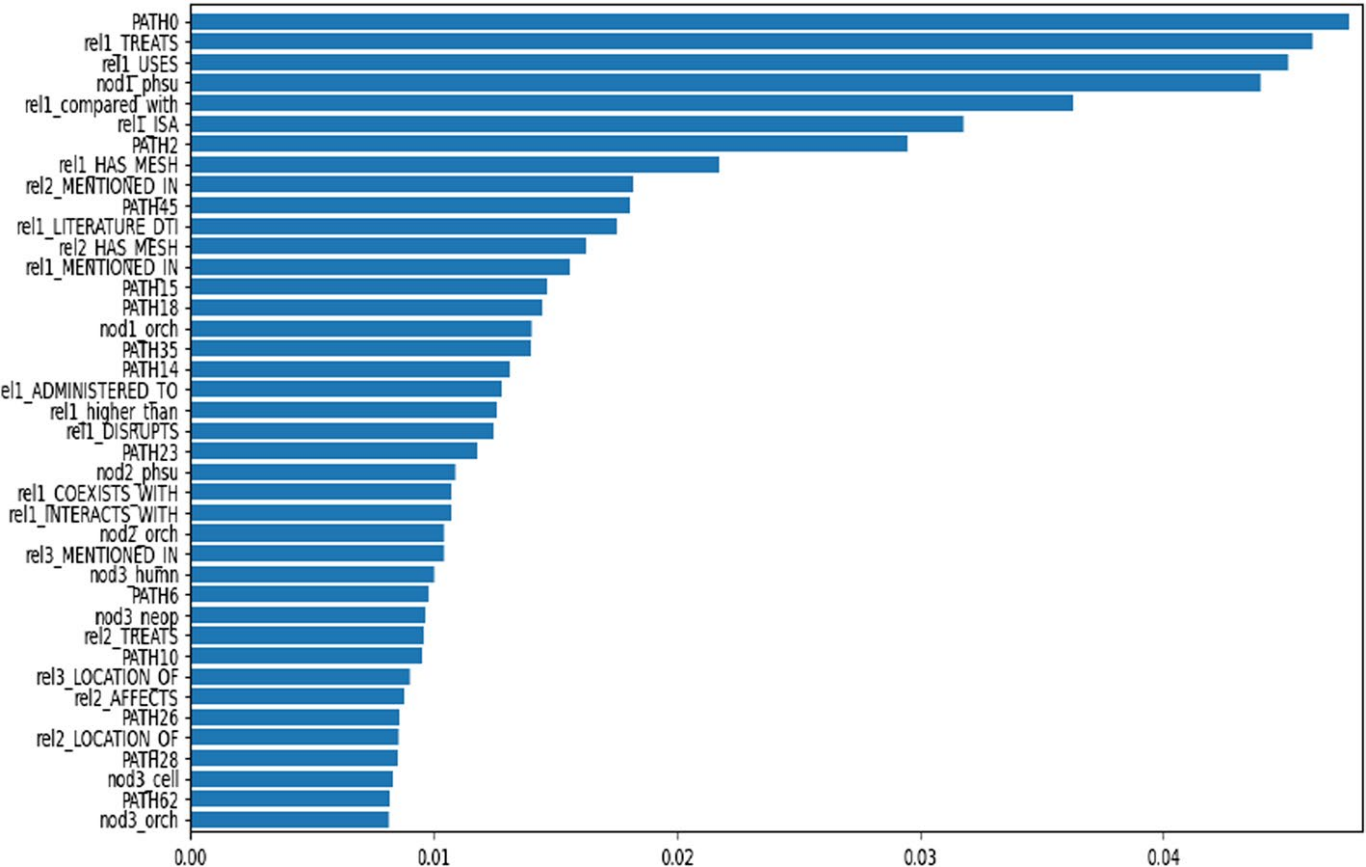
A list of the most important features and their importance scores, based on the decision forest used by BLGPA

BLGPA has the advantage of using features that can be interpreted, revealing important characteristics of the interacting drug-gene pairs. As a first step, Fig. 5 presents the 40 most important features used by BLGPA:

The selected features are mostly related with (a) the frequency of important paths between drug-gene pairs (e.g. PATH0 denoting the sequence of interaction relations (INTERACTS_WITH, INTERACTS_WITH, INTERACTS_WITH)), (b) frequent interactions (e.g. rel1_INTERACTS_WITH, rel1_LITERATURE_DTI), or (c) frequent treatment relations (e.g. rel1_TREATS) for the drug at hand. Case (c) implies that drugs frequently referred to as treatments in LC-related literature are more probable to be interacting with genes mentioned in such publications.

**Fig. 6:**
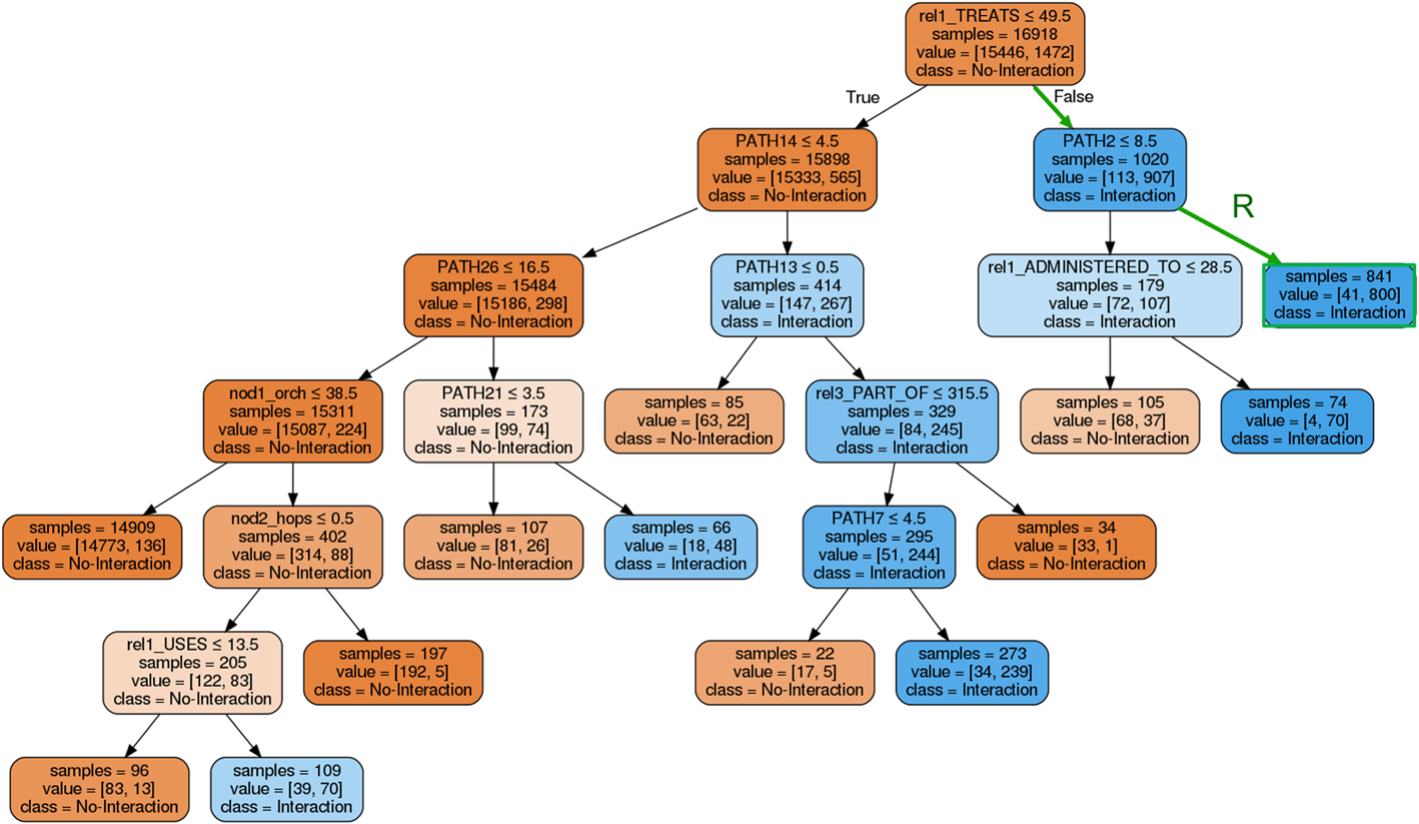
An approximation of the trained BLGPA model predictions by a decision tree. Illustration of an example rule R for classification

As an attempt to further explaining BLGPA model prediction results, a decision tree has been trained using the BLGPA output predictions on the dataset with the reduced 1:10 positive-to-negative ratio, taking advantage of the higher accuracy observed in lower imbalance cases. Figure 6 illustrates the resulting decision tree, using pre-pruning and GridSearch cross-validation for hyperparameter tuning,^12^ in order to avoid over-fitting. Additionally, an iterative post-pruning process was used to minimize depth and make the decision tree more concise. At each iteration, the accuracy and F1-score were calculated, to ensure that the resulting tree approximates BLGPA decisions accurately. The final decision tree (Fig. 6) approximates the Random Forest model with an accuracy of 0.98 and an F1-score of 0.87.

The path features used by BLGPA facilitate the explanation of predictions, following tree branches and related thresholds. As an indicative example, let us consider the Gefitinib drug (CUI: C1122962), used for the treatment of locally advanced or metastatic non-small cell Lung Cancer, and the ERBB3 Gene (CUI: C0812265). According to the NCI Thesaurus^13^ definition, Gefitinib inhibits the catalytic activity of numerous tyrosine kinases, including the epidermal growth factor receptor (EGFR), which may result in inhibition of tyrosine kinase-dependent tumor growth. Testing the interaction of Gefitinib with ERBB3, the path features extracted from the Knowledge Graph satisfy the conditions (1,2) shown in rule R of the decision tree, ending in a very pure class leaf (800/841 samples are positive):

Rule R: If *REL1_TREATS* $> 49.5$ AND *PATH2* $> 8.5$ THEN ‘Interaction’

Specifically, rule R imposes the following two requirements:

1. The drug (Gefitinib in our case) should be used frequently (50 or more cases in publications) for LC treatments connected to the target gene. Administration of Gefitinib for LC treatment actually appears in 3143 different publications.
2. PATH2 (TREATS $\rightarrow$ MENTIONED_IN $\rightarrow$ MENTIONED_IN) must appear more than 8 times between the two nodes (Gefitinib and ERBB3 in our case), meaning that the drug more frequently treats a symptom or condition that is co-mentioned with the target gene. The frequency of this path for Gefitinib and ERBB3 is actually 5333.

In a similar fashion, one can trace the rule applied to each individual drug-gene pair and explain the predictions of the decision tree. It should be stressed again that the tree is an approximation of the random forest, which is made possible due to the features being used by the latter.

### Drug rediscovery test

As a final test, we examined the potential use of DTI predictions on the popular task of drug rediscovery. In order to test the accuracy of the predictions, we generated the top-scoring predictions of the models and cross-checked them against online databases. In particular, we focused on drug-gene pairs that are highly ranked by each of the models (top 50), but are not in our dataset (i.e. considered as ‘False Positives’ in our evaluation). We then consulted a number of online repositories (SNAP, OpenTargets, bindingDB, STITCH, CTD, DrugCentral) that extract drug–target interactions automatically from various sources looking for those 50 pairs.

**Table 5:** Cross-validation of method predictions in other online databases. The last line highlights the number of predictions validated in any of the online databases for each method

Cross validation repository	BLGPA	RGCN	TransE	RESCAL	DistMult	HoLE
SNAP	1	2	1	0	2	1
OpenTargets	0	0	0	0	0	0
bindingDB	0	0	1	2	0	1
STITCH	18	11	15	4	11	7
CTD	19	11	14	8	10	14
DrugCentral	1	0	1	1	1	1
Any of these	**25**	**19**	**23**	**13**	**17**	**16**

As can be observed in Table 5, for BLGPA half of those predictions exist in one or more of the various online databases, while TransE follows closely. Despite the inherent uncertainty of such databases, this result is very promising, especially for predictions that appear in more than one repository. The detailed cross-validation results per pair can be found in the article’s Additional file 2.

## Conclusions

This paper presented and compared different link prediction approaches on a biomedical literature knowledge graph, on the task of drug–target interaction prediction. Based on the tests that we performed, graph embedding techniques seem to provide more accurate predictions than simple path analysis methods. However, a method proposed in the paper, which combines path ranking and semantic path features, achieves competitive results, while also facilitating the explainability of predictions. Furthermore, when applying the methods on a drug rediscovery test for lung cancer, we generated a number of candidates that were not present in the training data and could be verified against online databases. This is an indication that many DTIs are not present in our data and the models have achieved sufficient generalisation to be able to discover them. In addition to the comparison of the link prediction methods, we provide tools to map the identifiers of drugs and genes/proteins across various databases, and a benchmark of drug-gene interactions created from different online databases. As of future work, we plan to apply the various link prediction methods examined on a different disease-specific knowledge graph, in order to examine drug rediscovery for other disease use cases.

## Supplementary Information

**Additional file 1**. Appendix including the implementation details of each method as well as an overview of the hyper-parameters selected.

**Additional file 2**. The top-50 DTI predictions of every method and the cross-validation results per pair.

**Additional file 3**. A list of the 100 most interesting paths, identified by our Path Ranking method.

## Data Availability

The drug identifier mapping software is available in Github. The resulting mapping file can be downloaded from here. The protein/gene identifier mapping software is available in Github. The resulting mapping file can be downloaded from here The benchmark drug–target interaction dataset created from the union of Drugbank, KEGG, TTD and DGIdb is provided as a csv file. The BLGPA pipeline java code, as well as the python scripts for training and testing BLGPA, Graph Embeddings and RGCNs are available through the dedicated GitHub repository.

## Footnotes

1 https://github.com/fotais/drug-gene-interactions/blob/main/Full-DTIs-LC-Benchmark.csv.

2 http://db.idrblab.net/ttd/

3 https://www.ncbi.nlm.nih.gov/mesh/

4 https://neo4j.com/

5 https://lhncbc.nlm.nih.gov/ii/tools/MetaMap/Docs/SemanticTypes_2018AB.txt.

6 https://www.nlm.nih.gov/research/umls/META3_current_relations.html.

7 https://github.com/iit-Demokritos/drug_id_mapping.

8 https://github.com/iit-Demokritos/drug_id_mapping/blob/main/drug-mappings.tsv.

9 https://github.com/iit-Demokritos/target-id-mapping.

10 https://github.com/iit-Demokritos/target-id-mapping/blob/main/target-mappings_latest.tsv.

11 https://scikit-learn.org/stable/modules/generated/sklearn.feature_selection.SelectFromModel.html.

12 https://towardsdatascience.com/pre-pruning-or-post-pruning-1dbc8be5cb14.

13 https://ncithesaurus.nci.nih.gov/.
