# Supplementary material for: Comparing methods for drug–gene interaction prediction on the biomedical literature knowledge graph: performance versus explainability
Source: BMC Bioinformatics. 2023 Jun 30;24:272. doi: 10.1186/s12859-023-05373-2 (PMC10311852; doi:10.1186/s12859-023-05373-2)
Supplement: Supplementary file 1 — Additional file 1. Appendix including the implementation details of each method as well as an overview of the hyper-parameters selected. [file 12859_2023_5373_MOESM1_ESM.pdf]

Appendix

Implementation details

AnyBURL: AnyBURL requires as input a knowledge graph, in the form of tab separated values (tsv) files. Thus, the full Biomedical Literature Knowledge Graph had to be translated into a tsv format file. The groundtruth triples have been divided into ten folds through ten different files. For each repetition, the nine folds have been merged with the main knowledge graph tsv file, and one has been kept as testset. Using the prediction scores calculated by the AnyBURL algorithm, the source Java code of the tool has been edited in order to calculate the Precision, Recall and F1-Score metrics for each fold.

SemaTyP: We have re-implemented SemaTyP in Java, in order to make use of the Java API to the Neo4j database holding the KG. The Java SemaTyP implementation collects all DTD paths relating drugs with targets, and ignores article nodes, MENTIONED_IN relations, as well as triples retrieved only from a single article.

BLGPA: The path collection and SE+PR feature extraction modules have been also implemented in Java exploiting the Java API to the Neo4j database. The random forest classifier has been built in python using the scikit-learn[1] library.

Graph Embeddings: We have used the PyKEEN library[2] and PyTorch[3] to produce the TransE, DisMult, HoLE and RESCAL graph embeddings.

RGCN: For implementing the RGCN classifier, the KG had to be extracted in a tsv file, in order to build the Graph Convolution Network out of it. For this purpose, we have employed the RGCNEncoder of the PyTorch Geometric library[4].

Hyper-parameters

The following table provides an overview of the hyper-parameter values used in every method:

**AnyBURL**	• UN-SEEN NEGATIVE EXAMPLES=1<br>• TOP K OUTPUT=500<br>• THRESHOLD CORRECT PREDICTIONS=5<br>• SNAPSHOTS_AT = 5000
**SemaTyP**	• Maximum path lengths=3<br>• Logistic Regression model parameters:<br>◦ penalty=L2<br>◦ λ2=1.0<br>◦ solver='lbfgs'<br>◦ max_iter=13000

---

[1] https://scikit-learn.org/stable/modules/ensemble.html

[2] Ali, M., Berrendorf, M., Hoyt, C.T., Vermue, L., Sharifzadeh, S., Tresp, V., Lehmann, J.: PyKEEN 1.0: A Python Library for Training and Evaluating Knowledge Graph Embeddings. Journal of Machine Learning Research 22(82), 1–6 (2021)

[3] https://pytorch.org/

[4] https://pytorch-geometric.readthedocs.io/en/latest/

**BLGPA**	• Maximum path lengths=3<br>• Top-ranking paths=100<br>• Random Forest model parameters:<br>◦ no. of estimators=100<br>◦ criterion="gini"<br>◦ max_depth=None<br>◦ min_samples_split=2<br>◦ min_samples_leaf=1<br>◦ Feature Selection= SelectFromModel (threshold=0.003)
**TransE, DistMult, HoLE, RESCAL**	• Embedding size=100<br>• Max epoxhs=100 (early stop option)<br>• Random Forest model parameters:<br>◦ no. of estimators=100<br>◦ criterion="gini"<br>◦ max_depth=None<br>◦ min_samples_split=2<br>◦ min_samples_leaf=1
**RGCN**	• Encoder hidden layers=100<br>• Decoder=DistMult<br>• Optimizer = Adam optimization (learning_rate=0.01)<br>• Max epochs=15 / 50, (applied for 1:10 / 1:54 ratios respectively)
