# Supplementary material for: Comparing methods for drug–gene interaction prediction on the biomedical literature knowledge graph: performance versus explainability
Source: BMC Bioinformatics. 2023 Jun 30;24:272. doi: 10.1186/s12859-023-05373-2 (PMC10311852; doi:10.1186/s12859-023-05373-2)
Supplement: Supplementary file 3 — Additional file 3. A list of the 100 most interesting paths, identified by our Path Ranking method. [file 12859_2023_5373_MOESM3_ESM.docx]

1. compared_with,MENTIONED_IN,MENTIONED_IN
2. HAS_MESH,HAS_MESH,PART_OF
3. COEXISTS_WITH,MENTIONED_IN,MENTIONED_IN
4. HAS_MESH,MENTIONED_IN,LITERATURE_DTI
5. HAS_MESH,HAS_MESH,PRODUCES
6. HAS_MESH,HAS_MESH,AFFECTS
7. HAS_MESH,MENTIONED_IN,COEXISTS_WITH
8. HAS_MESH,MENTIONED_IN,PRODUCES
9. HAS_MESH,HAS_MESH,LITERATURE_DTI
10. HAS_MESH,HAS_MESH,TREATS
11. HAS_MESH,MENTIONED_IN,TREATS
12. HAS_MESH,HAS_MESH,PREDISPOSES
13. HAS_MESH,MENTIONED_IN,USES
14. HAS_MESH,MENTIONED_IN,compared_with
15. HAS_MESH,HAS_MESH,ASSOCIATED_WITH__SPEC__
16. HAS_MESH,MENTIONED_IN,STIMULATES
17. HAS_MESH,MENTIONED_IN,AFFECTS
18. LITERATURE_DTI,MENTIONED_IN,MENTIONED_IN
19. HAS_MESH,MENTIONED_IN,PART_OF
20. TREATS__SPEC__,MENTIONED_IN,MENTIONED_IN
21. MENTIONED_IN,MENTIONED_IN,LITERATURE_DTI
22. HAS_MESH,HAS_MESH,ASSOCIATED_WITH__INFER__
23. HAS_MESH,HAS_MESH,CAUSES
24. higher_than,MENTIONED_IN,MENTIONED_IN
25. HAS_MESH,HAS_MESH,STIMULATES
26. HAS_MESH,HAS_MESH,COEXISTS_WITH
27. HAS_MESH,MENTIONED_IN,INTERACTS_WITH
28. MENTIONED_IN,HAS_MESH,ASSOCIATED_WITH__SPEC__
29. HAS_MESH,MENTIONED_IN,ASSOCIATED_WITH__INFER__
30. TREATS__SPEC__,HAS_MESH,MENTIONED_IN
31. HAS_MESH,MENTIONED_IN,ASSOCIATED_WITH__SPEC__
32. HAS_MESH,MENTIONED_IN,INHIBITS
33. HAS_MESH,MENTIONED_IN,CAUSES
34. HAS_MESH,HAS_MESH,AUGMENTS
35. COEXISTS_WITH,HAS_MESH,MENTIONED_IN
36. HAS_MESH,MENTIONED_IN,ASSOCIATED_WITH
37. HAS_MESH,MENTIONED_IN,PREDISPOSES
38. HAS_MESH,HAS_MESH,INHIBITS
39. HAS_MESH,HAS_MESH,INTERACTS_WITH
40. MENTIONED_IN,HAS_MESH,LITERATURE_DTI
41. HAS_MESH,HAS_MESH,compared_with
42. MENTIONED_IN,HAS_MESH,PRODUCES
43. MENTIONED_IN,HAS_MESH,ASSOCIATED_WITH__INFER__
44. MENTIONED_IN,MENTIONED_IN,ASSOCIATED_WITH__SPEC__
45. HAS_MESH,MENTIONED_IN,PART_OF__SPEC__
46. compared_with,HAS_MESH,MENTIONED_IN
47. USES__SPEC__,MENTIONED_IN,MENTIONED_IN
48. MENTIONED_IN,HAS_MESH,COEXISTS_WITH
49. MENTIONED_IN,MENTIONED_IN,ASSOCIATED_WITH__INFER__
50. MENTIONED_IN,MENTIONED_IN,compared_with
51. MENTIONED_IN,HAS_MESH,STIMULATES
52. lower_than,MENTIONED_IN,MENTIONED_IN
53. HAS_MESH,HAS_MESH,ASSOCIATED_WITH
54. HAS_MESH,HAS_MESH,TREATS__SPEC__
55. COEXISTS_WITH__SPEC__,MENTIONED_IN,MENTIONED_IN
56. MENTIONED_IN,HAS_MESH,compared_with
57. STIMULATES,MENTIONED_IN,MENTIONED_IN
58. HAS_MESH,MENTIONED_IN,AUGMENTS
59. HAS_MESH,HAS_MESH,PART_OF__SPEC__
60. HAS_MESH,HAS_MESH,DISRUPTS
61. same_as,MENTIONED_IN,MENTIONED_IN
62. COEXISTS_WITH,INTERACTS_WITH,LITERATURE_DTI
63. INTERACTS_WITH__SPEC__,MENTIONED_IN,MENTIONED_IN
64. MENTIONED_IN,MENTIONED_IN,PART_OF__SPEC__
65. MENTIONED_IN,HAS_MESH,INHIBITS
66. MENTIONED_IN,HAS_MESH,INTERACTS_WITH
67. compared_with__SPEC__,MENTIONED_IN,MENTIONED_IN
68. DISRUPTS__SPEC__,HAS_MESH,MENTIONED_IN
69. COEXISTS_WITH,COEXISTS_WITH,LITERATURE_DTI
70. compared_with,COEXISTS_WITH,COEXISTS_WITH
71. compared_with,INTERACTS_WITH,STIMULATES
72. MENTIONED_IN,HAS_MESH,TREATS__SPEC__
73. INHIBITS,HAS_MESH,MENTIONED_IN
74. COEXISTS_WITH,COEXISTS_WITH,COEXISTS_WITH
75. STIMULATES,HAS_MESH,MENTIONED_IN
76. INHIBITS__SPEC__,MENTIONED_IN,MENTIONED_IN
77. compared_with,INTERACTS_WITH,LITERATURE_DTI
78. compared_with,COEXISTS_WITH,STIMULATES
79. HAS_MESH,MENTIONED_IN,PRODUCES__SPEC__
80. COEXISTS_WITH,INTERACTS_WITH,STIMULATES
81. HAS_MESH,HAS_MESH,PRODUCES__SPEC__
82. higher_than,HAS_MESH,MENTIONED_IN
83. compared_with,INTERACTS_WITH,COEXISTS_WITH
84. COEXISTS_WITH,INTERACTS_WITH,COEXISTS_WITH
85. HAS_MESH,HAS_MESH,DISRUPTS__SPEC__
86. compared_with,COEXISTS_WITH,LITERATURE_DTI
87. compared_with,compared_with,LITERATURE_DTI
88. compared_with,compared_with,COEXISTS_WITH
89. ASSOCIATED_WITH__INFER__,MENTIONED_IN,MENTIONED_IN
90. compared_with,TREATS,ASSOCIATED_WITH
91. MENTIONED_IN,HAS_MESH,PRODUCES__SPEC__
92. MENTIONED_IN,HAS_MESH,PART_OF__SPEC__
93. COEXISTS_WITH__SPEC__,HAS_MESH,MENTIONED_IN
94. compared_with,TREATS,PART_OF
95. compared_with,INTERACTS_WITH,INTERACTS_WITH
96. MENTIONED_IN,MENTIONED_IN,PRODUCES__SPEC__
97. COEXISTS_WITH,COEXISTS_WITH,STIMULATES
98. HAS_MESH,MENTIONED_IN,higher_than
99. INTERACTS_WITH__SPEC__,HAS_MESH,MENTIONED_IN
100. compared_with,INTERACTS_WITH,INHIBITS
